# A systematic review and meta-analysis on the main purpose of diagnostic classification systems and their utility for various purposes

**DOI:** 10.1192/j.eurpsy.2023.219

**Published:** 2023-07-19

**Authors:** E. Vrigkou, R. Stamatakis, K. Umla-Runge

**Affiliations:** 1 Centre for Medical Education, Cardiff University, Cardiff; 2 The Caswell Clinic, Swansea Bay, United Kingdom

## Abstract

**Introduction:**

The development of the 11th edition of the International Classification of Diseases has rekindled the research interest surrounding diagnostic classification systems (DCSs). According to expert consensus, the main purpose of DCSs is to provide guidance in clinical practice. There are no reviews in the international literature, however, assessing what mental health practitioners believe is the main purpose of DCSs.

**Objectives:**

The aims of this systematic review were to assess what mental health professionals think is the single most important purpose of DCSs and how they rate DCSs’ utility for various purposes. All DCSs were considered.

**Methods:**

Two separate searches were conducted in Medline Via Ovid and PsycInfo: one for articles assessing the main purpose of DCSs according to mental health professionals and one for studies on how practitioners rate the utility of DCSs for various purposes. The first search revealed eight articles on the main purpose of DCSs and the second three articles on how practitioners rate their utility for various purposes. The total number of participants from all included studies for the first search was 9,276 and for the second 2,363. The studies included clinicians from a wide range of world regions, languages, and income-level countries.

**Results:**

The results of the meta-analyses for the first search showed that 44% (95%CI=38-49%) of the responders believe that the main purpose of the DCSs is facilitating inter-clinician communication, 20% (4-35%) to inform treatment decisions, 14% (11-16%) to aid the communication between clinicians and patients, 11% (4-18%) to reflect on aetiology/pathogenesis, 9% (2-16%) to facilitate research, 4% (2-7%) to provide a national statistical base and 1% (0.1-2%) to indicate prognosis. Regarding how responders rate the utility of DCSs for various purposes, the highest ratings were given for meeting administrative requirements and inter-clinician communication in the two of the three included studies, and clinical diagnosis and training in the third.

**Conclusions:**

“Inter-clinician communication” was the most voted purpose of DCSs and was rated relatively high in the tier of DCSs’ clinical utility. In contrast, “inform management decisions”, even though it was voted as the second most popular purpose of DCSs, was placed on the bottom of the rating tier of DCSs’ clinical utility. Interestingly, none of the included studies asked the responders whether “making a diagnosis” is the main purpose of DCSs. Further research is needed to assess what mental health professionals expect from DCSs, so as to improve their clinical utility in the future.

**Disclosure of Interest:**

None Declared

